# Perceptions of private specialist outreach services at a rural district hospital, South Africa

**DOI:** 10.4102/safp.v65i1.5641

**Published:** 2023-01-23

**Authors:** Hayden L. Poulter, Louis S. Jenkins, Paul A. Kapp

**Affiliations:** 1 Department of Family and Emergency Medicine, Faculty of Medicine and Health Sciences, Stellenbosch University, Cape Town, South Africa; 2 Department of Family and Emergency Medicine, Knysna Provincial Hospital, Western Cape Department of Health, Knysna, South Africa; 3 Primary Health Care Directorate, Department of Family, Emergency and Community Medicine, Faculty of Health Sciences, University of Cape Town, Cape Town, South Africa; 4 Department of Family and Emergency Medicine, George Hospital, Western Cape Department of Health, George, South Africa

**Keywords:** private specialists, outreach, rural, district hospital, perceptions

## Abstract

**Background:**

A major disparity exists in access to specialised healthcare between rural and urban areas. Specialist outreach programmes are one of the ways in which rural specialist healthcare inequality is being addressed. A number of rural district hospitals (RDH) employ local, private specialists (LPS) to supplement public specialist outreach. Limited research exists on private specialist outreach and support (PSOS) in sub-Saharan Africa or South Africa.

**Methods:**

This was a descriptive, exploratory, qualitative study using thematic analysis of semi-structured interviews. Non-probability, purposive sampling was used to obtain a sample size of 16 participants. The audio recordings were transcribed verbatim and analysed with the framework method and ATLAS.ti version 8^©^ software.

**Results:**

Four major themes emerged, namely roles of LPS, effects, sustainability and feasibility of PSOS. Overall PSOS was considered sustainable, feasible and had positive effects in and beyond the sub-districts. The value of PSOS was supported by improved access and timeliness of services, improved competency of RDH medical practitioners, improved coordination, comprehensiveness and continuity of care. Private specialist outreach and support was, however, associated with increased burden on the RDH resources and required a basic level of RDH infrastructure to function effectively.

**Conclusion:**

The perceived contribution of private specialist outreach services was positive overall. Implementation in RDHs is feasible, but should involve consideration of factors in the hospital, town, sub-district and district prior to implementation.

**Contribution:**

This paper provides evidence that private specialist outreach and support services are feasible in the state health sector, provided that certain considerations are taken into account.

## Background

Almost half of South Africa’s population live in rural areas with only 12% of doctors providing care to these patients.^1^ Consequently, rural specialist healthcare services are very limited.^2,3^ Specialist outreach programmes are one of the ways in which rural specialist healthcare inequality is being addressed, both globally and locally.^2,3^ These programmes involve specialists based in urban settings travelling to rural areas to provide specialist outreach and support.^2,3,4,5,6^

Multifaceted specialist outreach focuses not only on clinical care but also includes capacity building and clinical governance. It has been shown to improve health outcomes and access to care, provide more efficient and evidence-based care, and reduce hospitalisations and congestion at regional and tertiary hospitals.^4,5,7,8,9,10^ A number of factors have been identified that negatively influence public sector outreach, resulting in suboptimal outreach and support to rural settings.^3,9^

A number of rural district hospitals (RDHs) in the Western Cape and in the rest of South Africa employ local, private specialists (LPS) on a part-time or sessional basis to supplement public specialist outreach.^5^ Knysna Provincial Hospital (KPH) is one of those facilities. Private specialist outreach and support (PSOS) in the public sector can assist with improved geographic access to specialist care, reduced demand for patient transfer and reduced theatre time pressures for elective surgery at regional hospitals.^4,5,8^

Very little published research exists on PSOS in South Africa, and no research on PSOS in the Garden Route district regarding the roles, effects, sustainability and feasibility of this service exists.^11^ Exploring local PSOS would provide new knowledge to the field of rural healthcare. The aim of this research was to explore the perceived contribution of PSOS to healthcare services at a RDH in the Garden Route district of South Africa. The objectives included exploring the roles of local private specialists in PSOS, and the effects, sustainability and feasibility of PSOS.

## Methods

### Study design

This was a descriptive, exploratory and qualitative study.

### Setting

The study was based in the rural setting of the Knysna and Bitou sub-districts of the Garden Route district, Western Cape. The total population of both sub-districts was approximately 134 000.^12,13^ Knysna Provincial Hospital is a 90-bed district hospital and the only public hospital in both sub-districts. There are 13 day-clinics in both sub-districts. The hospital consists of a male, female, paediatric and maternity ward with outpatient services, an emergency centre, two operating theatres and a rehabilitation centre. There is a daytime, on-site laboratory service and a full time X-ray service. George regional hospital (GRH) is the referral hospital, 65 km away, 1 h by road transport (see Figure 1^14^). The tertiary referral hospital is 6 h away by road transport. Most specialist departments at GRH provide some outreach and support to KPH. Public specialist outreach has been considerably negatively impacted by the coronavirus disease 2019 (COVID-19) pandemic because of resource and staff constraints. Before the COVID-19 pandemic, obstetrics and gynaecology and internal medicine provided weekly outreach, family medicine and psychiatry provided monthly outreach, while surgery, paediatrics and orthopaedics provided outreach every few months. Anaesthetics provided no outreach. Knysna Provincial Hospital has five LPS who provide PSOS, two who are employed and three who work pro bono. While public services outreaches competed with many variables, including travel distances, local service pressures, and staffing constraints, PSOS, on the other hand, were close by (in the same town), more flexible to adjust to immediate changes in service routines, and tended to develop closer professional relationships over time.

**FIGURE 1:**
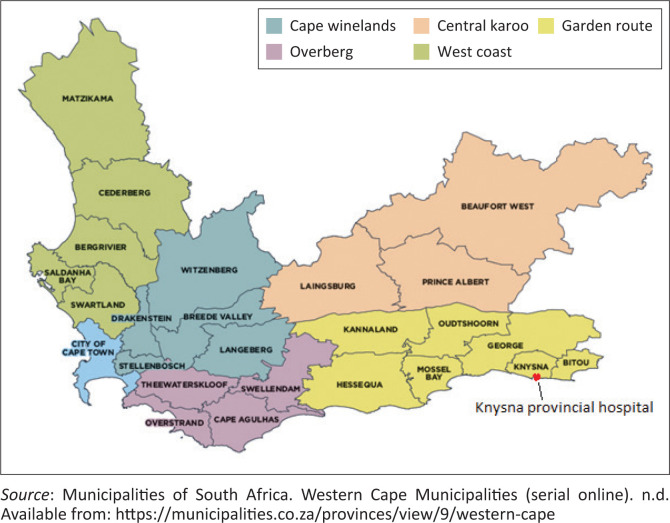
A regional representation of the Western Cape demonstrating the location of the Knysna and Bitou sub-districts in relation to George sub-district, the rest of the Garden Route district and Cape Town.

### Study population and sampling

The study population included private and public specialists, managers, family physicians, medical officers and family medicine registrars associated with the hospital and sub-district. Non-probability, purposive sampling was used to obtain a sample size of 16 participants, at which point data saturation was reached, because no new themes were emerging during the interviews. Four categories of stakeholders were selected, namely managers, family physicians, medical officers and registrars and specialists. These categories were chosen in order to allow wide representation and hopefully would provide rich information. Only stakeholders who had been working in the sub-districts for more than six months were included.

### Data collection

Individual, semi-structured interviews took place using an interview guide during February 2021 and March 2021 (see Appendix 1). These were conducted by the principal researcher and were arranged in advance. The interviews with the managers, medical officers, registrars and some of the specialists took place in a private room at KPH. The remainder of the specialists were interviewed in their consultation rooms at their private practices. The interviews were recorded on two audio recording devices. The interviews were saved and stored securely on two hard drives, on two separate computers with password protection.

### Data analysis

The audio recordings were transcribed verbatim using a professional company, approved by the ethics committee for *Protection of Personal Information Act* (POPI) compliance. Personal identifiers were not used in the analysis and reporting. The data were analysed using the framework approach^15^ with the support of ATLAS.ti software version 8^©^. The steps were as follows:

- Familiarisation occurred through listening to the audio recordings of the interviews, reviewing field notes and reading the transcripts.
- A list of codes and categories were developed inductively, while keeping the objectives of the study in mind (deductively).
- From this list, a thematic index was created.
- The codes in the thematic index were applied systematically to all the transcripts.
- Categories and codes were charted, in order to cluster data from the same categories.

### Trustworthiness

Guba’s model for assessing the trustworthiness of the data was applied.^16^ The credibility of the research was enhanced by using various interviewing techniques, including listening, observation, probing, restating and summarising. Respondents validated the interpretations, which further enhanced the credibility of the findings. By describing the research methods in detail, a degree of transferability was achieved. The transcribed audio recordings were checked for any missing data against the audio recordings by the researchers and a moderator to improve trustworthiness. Data were read and re-read for familiarisation by two independent researchers, who helped to supervise the research, to obtain an in-depth understanding of its content. The researchers discussed the themes at length until a consensus was reached. Significant quotes were highlighted, and patterns were coded. An expert in qualitative research, employed at a tertiary institution, was used to moderate codes independently. Triangulation with South African and international literature, member checking and field notes allowed for confirmability. A clear audit trail was maintained from tape to transcript to analysis in ATLAS.ti software version 8^©^ (Scientific Software Development, Berlin, Germany). Finally, reflexive practice by the principal researcher helped to minimise personal biases.

### Ethical considerations

Ethics approval was granted by the Health Research Ethics Committee of the University of Stellenbosch (S20/06/140). Permission was granted by the Research Committee of the Department of Health of the Western Cape (WC_202010_050), the district manager, and district hospital management. Voluntary informed consent was obtained from the respondents.

## Results

Sixteen semi-structured interviews were conducted. The mean interview time was 44 min. An equal number of male and female participants were interviewed, with a mean age of 42 years (see Table 1). To maintain participant confidentiality, public and private specialists were not subdivided further.

**TABLE 1:** Demographics of study participants.

Demographics of participants	Number
Number of public and private specialists	7
Management (hospital and district)	3
Family physicians	2
Medical officers and/or family medicine registrars	4
Age range (years)	28–61

Four major themes emerged, namely the roles of LPS, the effects of PSOS, the sustainability of PSOS and the feasibility of PSOS. Within these, several minor themes were identified (see Table 2).

**TABLE 2:** The major and minor themes.

Themes	Major	Minor
1	Roles of local private specialists	Clinical consultant
		Capacity builder
		Clinical governance
2	Effects of PSOS	Benefits for the local community
		Effects on medical practitioners
		Effects beyond the sub-district
3	Sustainability of PSOS	The benefits to LPS
		Public–private partnerships
		Employed versus pro bono LPS
		Public versus PSOS
4	Feasibility of PSOS	Financial implications of PSOS
		Resource and infrastructure requirements
		Implementation of PSOS in other RDHs.

PSOS, private specialist outreach and support; LPS, local, private specialists; RDH, rural district hospitals.

### Roles of local private specialists

The roles of LPS providing PSOS were captured within three minor themes: clinical consultant, capacity builder and clinical governance.

#### Clinical consultant

Outpatient care, ward rounds, theatre work, consultations and training were clinical services provided by LPS. Of these, outpatient care, theatre work and telephonic consultations were the most important. The needs of the RDH guided these clinical services. Local, private specialists were not involved in general ward rounds. Ward rounds were performed on an individual patient basis, where a specific problem or postoperative patient review was needed. This was the most efficient way of maximising the LPS’s time during PSOS. Postoperative care plans and postoperative emergencies were the responsibility of the LPS.

Digital and telephonic consultations with LPS were important services. These consultations were conducted via email and telephonic communication channels. Communication with LPS was easier than with their public counterparts. Direct communication with LPS avoided patient discussions with junior practitioners at the referral hospital. This resulted in improved patient management:

‘I think the benefit to being a specialist is that when you are contacted, there’s the anticipation on the other side that you are speaking to a very highly qualified individual with a very specific language, that you can tap into. There’s no iffyness of speaking to someone with less qualification and faffing around to get to an answer. You are a person with a vast wealth of information, that has immediate benefit.’ (Public specialist 1)

The RDH PSOS ‘champion’ or liaison doctor was crucial to an efficient PSOS clinical service programme. The term RDH ‘champion’ was used by many participants. It referred to the medical practitioner from the RDH who worked side-by-side with the LPS. They were responsible for the administration of the PSOS day, liaising with the LPS and accompanying the LPS while PSOS took place, to assist and learn.

#### Capacity builder

The role as a capacity builder was the most dominant, non-clinical role of the LPS, contributing greatly to the value of PSOS. Capacity building took place through skills transfer during in-person and digital patient discussions, tutorials and presentations. The main beneficiaries of skills transfer were the RDH ‘champions’. The level of skills transferred was dependent on the recipient’s attentiveness and willingness to learn:

‘The private specialists provide good teaching opportunities and training wherever they are. If someone is around them and they’re prepared to observe and listen, they’re going to get a lot out of the specialist, because there’s a concentration of skills and knowledge that you would not necessarily otherwise have, unless you are a specialist.’ (Public specialist 1)

Capacity building was performed formally and informally. The formality of capacity building was dependent on the LPS preferences. Most LPS were keen to teach and train. Public multi-faceted specialist outreach was observed by some participants to involve little or no capacity building. Some of the LPS participants stated that they were from academic backgrounds, where a culture of teaching and learning existed. Private specialist outreach and support afforded the LPS the opportunity to continue with this practice. Local private specialists attended conferences in their specialty as part of their continuing professional development and brought the knowledge gained into the RDH working environment. This contributed to improving the quality of service and helped to maintain up-to-date, evidence-based practises.

Skills transfer and capacity building were dependent on the RDH teaching and learning culture. The local hospital was observed to have an ‘ethos of learning and upskilling’ and therefore capacity building was effective. The bedside teaching during PSOS had a knock-on effect of educating the patients on their conditions by engaging them in the discussion between LPS and learners.

One participant was concerned with the discrepancy in the way in which the public and private sectors practiced. Public practice was considered by this participant to be more guideline-based, while private practice was more reliant on individual preference. While this potentially negatively affected the capacity building programme, it could also offer an opportunity for synergism:

‘Some of us like the academic environment, we like the teaching, we like learning. And we would have loved to have stayed in an academic environment but an academic environment is limited, it doesn’t have a big structure to hold all of us. So we have to move out. But this role, this outreach gives those, that sector of doctor an opportunity to come back in. And there is definitely a win-win situation.’ (Private specialist 3)

Many junior doctors mostly experienced exposure to specialties in secondary or tertiary hospitals. Private specialist outreach and support afforded junior doctors exposure to specialties and LPS who live and work in a rural town and a RDH. This allowed them to gain perspectives that may have influenced their chosen career paths.

The majority of participants felt that the availability of RDH medical practitioners was not negatively affected by PSOS. A small number of participants felt the opposite, namely that RDH medical practioner availability was negatively affected, which also occurred with public specialist outreach:

‘So they [*specialists*] can’t function without help [*an assistant*]. And sometimes when we are short-staffed, that’s a problem. It’s generally not though.’ (Medical officer 3)

#### Clinical governance

Participants had mixed feelings about LPS involvement in clinical governance at the RDH (see Table 3). There were mixed opinions within the groups. For example, some private specialists felt that the focus should be on service provision and capacity building, while others felt that time was limited and therefore the focus should be on service provision and wanted to be involved in clinical governance. While most LPS emphasised good quality of care in PSOS, they were not directly involved in any quality improvement projects or morbidity and mortality meetings.

**TABLE 3:** Factors for and against local, private specialists involvement in clinical governance at rural district hospitals.

Factors for LPS involvement	Factors against LPS involvement
Provide innovative ways of thinking	Need to have deep understanding of public healthcare
Gives an objective view of the case/problem	Need to understand what resources are available
Improves standards of care and expectations	Should not be able to make changes to the system
Offers alternate solutions to public doctors	Local, private specialists have different budgets and priorities in private
There is a cross-pollination of ideas	Time is better spent doing teaching and training
Already involved in improving quality of care, but not on a formal basis	Can offer advice, but there is a limit to what can be changed

LPS, local, private specialists; RDH, rural district hospitals.

### Effects of private specialist outreach and support

Three minor themes emerged: Benefits for the local community, effects on medical practitioners and effects beyond the sub-district.

#### Benefits for the local community

It was unanimous that access to services for the local community was improved. This positive effect was greatest where transport between the RDH and secondary or tertiary referral hospital was associated with longer travel times. Factors linked with improved access to care were better timeliness of services and geographic access, and financial benefits for the health system and the patient. Some PSOS services were inaccessible or unavailable in the public sector, such as plastic surgery.

By reducing the demand for patient transfer, PSOS improved the capacity for transportation of other patients from the RDH to the referral centres and vice versa. Lifesaving surgical intervention performed by the LPS surgeon on patients too unstable for transfer had saved numerous lives since PSOS started:

‘I’m talking specifically about surgery as we have had a number of occasions where somebody has a stabbed heart and would never have survived without our private surgeon saying phone me any time I will come. So I think the effects and value in that way are actually incalculable.’ (Family physician 1)

Improved access and timeliness of services reduced the likelihood of loss to follow up and development of complications of untreated conditions. Fewer complications resulted in less cost to the state in long term, lower morbidity and mortality, improved quality of life for the patient, less income loss and reduced loss to the family of the patient and their community. Overall, the coordination of care was improved. Table 4 illustrates PSOS factors for and against improved RDH coordination of care:

‘You start to learn how that other person thinks. You start to learn what they’re [*local, private specialists*] going to be looking for in this patient before you refer. I think by observation and being in their presence you’re going to pick up an awful lot that’s going to streamline the service.’ (Public specialist 1)

**TABLE 4:** Private specialist outreach and support factors for and against improved rural district hospitals coordination of care.

For improved RDH coordination of care	Against improved RDH coordination of care
The RDH ‘champion’ liaises between RDH and LPS to coordinate care and streamline the service.	The PSOS can result in backpressure and resistance from the referral centres to referring patients in the LPS specialty domain because of the assumption that the LPS can manage those patients instead.
Relationship with LPS results in less resistance to referral or advice, compared with the public sector counterparts.	Referral of patients, seen by the LPS in the RDH, to the referral centres require the same referral pathway as other patients.
Direct communication with the LPS, as opposed to a junior medical practitioner at the referral centre, results in improved patient management coordination.	
Some of the LPS have direct contact with specialists working at tertiary referral centres.	
The LPS ‘triages’ and ‘lubricates’ the referral of patients to referral centres.	
Open communication channels with LPS enabled timely clinic patient management resulting in fewer outpatient visits and referrals to the district hospital.	

PSOS, private specialist outreach and support; LPS, local, private specialists; RDH, rural district hospitals

There was unanimous agreement that the comprehensiveness of care was improved by PSOS. Improved comprehensiveness of care was linked to offering a variety of PSOS disciplines, RDH staff upskilling, and specialist care management without the need for referral:

‘If you have specialists you can deliver a bigger package of care so it’s a no-brainer for me that your care package becomes much more comprehensive.’ (Manager 3)

Participants perceived that continuity of care was improved by PSOS. The following reasons were identified: The LPS providing PSOS did not change on a weekly or monthly basis as what commonly occurred with the public counterparts; LPS were accustomed to following up their own patients in private practice and did so in PSOS; the RDH ‘champions’ for PSOS services remained the same for several months, which improved continuity of care; the RDH ‘champion’ became the regular, primary service provider for some patients once certain skills were attained; outpatient, perioperative and follow-up care was provided by the same LPS providing PSOS; more timely service with PSOS resulted in fewer patients lost to follow up:

‘Without a doubt it improves continuity of care. If you see the same specialist multiple times, they start to get to know your history, your complaints, your issues, so absolutely.’ (Manager 3)

#### Effects on medical practitioners

Rural district hospitals medical practitioners felt that their clinical competencies improved. The long-term positive impact of upskilled RDH medical practitioners was considered the most notable effect of PSOS. This enabled RDH medical practitioners to train their colleagues in the RDH, improving the competency of other RDH staff. Regular rotation of the RDH ‘champion’ every 6 to 12 months also resulted in a greater number of upskilled staff. Local, private specialists provided expertise in their field and reduced the likelihood of complacency in RDH staff:

‘We started with some very basic simple disorders within my speciality where the knowledge was really lacking. But once I had done two or three months of teaching, I could already see that those patients were being dealt with at the outpatients without referral to me because the doctors felt competent, they felt knowledgeable, they felt they could go out and do it.’ (Private specialist 6)

Private specialist outreach and support generated ‘positivity and optimism’ which resulted in ‘enrichment and growth’. Local, private specialists were morale boosting. The presence of a LPS had a knock-on effect encouraging staff to ‘try harder and be better’. This affected clinical, management and administration staff. Non-clinical staff aimed to ensure that things were in place and ran smoothly:

‘And I think we underestimate what it means to be happy at your work. And one of the things that can make one happy at work is knowing we’ve got this guy, or this girl and they are awesome people, great specialists, they deliver an amazing service and I’m going to be with them today, yes. It’s going to be a good day because I’m going to learn stuff.’ (Public specialist 1)

#### Effects beyond the sub-district

Private specialist outreach and support had a positive effect on the district and referral hospital. The most dominant effect on the district was the reduced burden on the referral hospital from the RDH. Private specialist outreach and support was also found to reduce the burden on the tertiary referral centres. For example, plastic surgery on the face under local anaesthetic and specialised dermatological services was performed during PSOS at the RDH. These alleviated burdens included reduced time and financial contraints, reduced bed pressure and decreased theatre-time pressures. This allowed the referral hospitals to focus on patient referrals from the RDH that were not appropriate for district hospital care, and referrals from the rest of the district. Referrals considered not appropriate for RDH care were those requiring intensive postoperative care, high-risk surgery, and those that required a specialist anaesthetist:

‘I mean you cannot ignore the fact that what happens here in this hospital is going to have an effect on the district. The ripple effects of having an efficient system here means that a place like George hospital can have less worries about what happens at its Eastern District Hospital at Knysna because there’s trust in the efficiency of the service, the competency of the service, the speed at which the service can be called upon in dire emergencies because they’re [*local, private specialists*] close by and they’re willing. And that has an effect on emergency care. Management doesn’t have to worry about these sorts of things because if a stab heart comes in well, he’s willing, he comes, he’s quick. That saves a life.’ (Public specialist 1)

Decentralisation of specialist care was observed by several participants to be an important healthcare system process. Private specialist outreach and support facilitated this process. It was felt that district, provincial and national government should put more emphasis on decentralisation of healthcare with PSOS:

‘So if the country as a whole are looking at how to save money for people, then it’s probably to bring the service to the people rather than centralising services in big centres.’ (Manager 3)

Private specialist outreach and support at KPH resulted in positive engagement of other sub-districts in the Garden Route. Doctors from other sub-districts regularly consulted with and referred patients to PSOS at KPH. Doctors from other sub-districts occasionally attended capacity building opportunities at KPH. Medical practitioners who were employed by and upskilled at KPH, relocated to other RDH in the district with improved competency. Private specialist outreach and support improved positivity and optimism in the district:

‘There are patients in other sub-districts that benefit from private specialist outreach, which is quite remarkable.’ (Medical officer 1)

### Sustainability of private specialist outreach and support

The sustainability of PSOS was captured within four minor themes, namely the benefits to LPS, public–private partnerships, employed versus pro bono LPS, and public versus PSOS.

#### The benefits to local, private specialists

Private specialist outreach and support benefitted the LPS, who were considered to have a mutually beneficial relationship with the RDH. This relationship contributed positively towards the sustainability of PSOS. Multiple benefits were identified. Some were observed by the majority of participants, while others were noticed by only a few. These were divided into dominant and non-dominant benefits, respectively (see Table 5):

‘In private you get into almost like a rut where you work, and you work, and you work, and your patients are very very demanding and there’s hardly ever a thank you and that can become a bit demoralising. So when you do go to the public sector you feel that you’re actually making a difference in someone’s life.’ (Private specialist 3)

**TABLE 5:** Dominant and non-dominant benefits of private specialist outreach and support to local, private specialists.

Dominant benefits	Non-dominant benefits
Felt part of a team	Accumulated continuous professional development points
Sense of belonging to the RDH and felt appreciated	Broke the monotony of private practice
Improved continuity of care for LPS private≈patients no longer able to afford private healthcare	Learned from the RDH medical practitioners about other specialties and resource management
Exposure of LPS to stimulating pathology	Satisfied the need to contribute to the community
Fulfilled the need for LPS academic teaching	Earned extra salary
	Streamlined referrals of private patients into the RDH healthcare network by understanding the referral pathway
	Local, general practitioners provide preferential referral of their cash-based private patients to the LPS’s private practice, because of the potential of providing further care via the private–public referral pathway, if needed
	Preferential referral to the LPS private practice from local, general practitioners who were previously employed at the RDH where PSOS took place
	The RDH staff referred their family members and friends to the private practice of the LPS providing PSOS

PSOS, private specialist outreach and support; LPS, local, private specialists; RDH, rural district hospitals.

#### Public–private partnerships

This partnership improved referrals between the private and public sector and vice versa. The value of this relationship was important in a small town where possibly more public–private collaboration took place compared with larger towns or cities. Private specialist outreach and support improved the public–private partnership and allowed improved access to infrastructure, resources and pharmaceuticals for both parties. These relationships were even more important with National Health Insurance on the horizon.

All participants felt that the personal and professional relationships that formed between RDH staff and LPS positively contributed to the sustainability of the service. One participant felt that PSOS was ‘almost entirely relationship driven’. During PSOS, LPS functioned as part of the RDH team, in contrast to their private practice. This contributed to making the LPS feel appreciated and further strengthened the relationship:

‘Private specialists are a valuable link between the private sector and the public sector, not only for patient care, but also for relationships between colleagues, and yes generally in a small town creating the sense that it’s not separate.’ (Family physician 1)

There was improved sustainability with the involvement of the RDH managers in the PSOS public–private partnership. Management improved sustainability by engagement in PSOS and fostered positive relationships with LPS:

‘But I think the people at this hospital, and the managerial side have worked hard and long with their specialists to really have a great relationship with them, and I think they’ve crafted sustainability into this whole mix.’ (Public specialist 1)

#### Employed versus pro bono local, private specialists

Most participants felt that LPS who were employed, as opposed to working pro bono, may provide a more sustainable service. Although no pro bono LPS had terminated their PSOS by the time this research took place, the lack of an employment contract could make terminating pro bono PSOS easier compared with their employed counterparts. The sustainability depended more on the LPS’ reasons for involvement in PSOS, than their employment status. One participant felt that all LPS providing PSOS should be employed. Several factors were identified that may contribute to LPS-PSOS termination. These factors applied more to pro bono LPS, namely increasing private practice workload, poorly functioning RDH health systems with resultant frustration, LPS relocating, and being overworked by the RDH:

‘Look, everybody has their price hey. And you might do it pro bono, but your payment comes in the emotional reward of having a good day because things work and people are friendly and service works. I think once those things are not working then if you don’t get money for it then you lose the impetus to do it.’ (Public specialist 1)

#### Public versus private specialist outreach and support

The majority of participants felt that public specialist outreach was more sustainable than PSOS long term. However, PSOS was considered more sustainable than public specialist outreach over the short term during the COVID-19 pandemic. Public specialist outreach formed part of the job description of public specialists, and was the responsibility of the referral hospital. A small number of participants felt that some public specialists were reluctant to do outreach.

This contributed to animosity between the public specialist and the RDH. Some public specialists were pro-outreach and this fostered good working relationships:

‘It is forced. It has to be part of the process that the regional hospitals provide outreach. So whether this person (public specialist) wants to give outreach or not, if they change, if there’s a new person or whatever, you’re not going to suddenly halt the service, it will continue to be provided, unless there’s a pandemic.’ (Family physician 2)

### Feasibility of private specialist outreach and support

Three minor themes emerged when the feasibility of PSOS was explored. These were financial implications of PSOS, resource and infrastructure requirements and implementation of PSOS in other RDHs.

#### Financial implications of private specialist outreach and support

The majority of participants felt that there were financial benefits to having PSOS, both for the healthcare system and the patients (see Table 6).

**TABLE 6:** The financial benefits of private specialist outreach and support.

Healthcare system factors	Patient factors
Reduced transport costs to and from the referral centres (fuel, maintenance of the ambulance, ambulance crew)	Less time away from work and loss of income
Relationships with LPS resulted in private funding and donations from private welfare organisation	Less likely to seek private healthcare because of timely treatment
Reduced need to employ additional public specialists as PSOS reduced referrals	Less morbidity and mortailty and therefore less loss of income
Lowered risk of complications from underlying condition because of timely treatment, resulting in lower long-term cost to the healthcare system	Patients and parents incur accommodation costs when referred to tertiary referral centres in Cape Town. Treatment at the RDH by LPS alleviates this cost.
Shorter hospital stays at RDH compared with the referral centres	-

PSOS, private specialist outreach and support; LPS, local, private specialists; RDH, rural district hospitals.

Several participants observed that saving transport costs would not benefit the RDH budget directly, but would positively effect provincial budget. The biggest benefit would occur with PSOS tailored to the greatest needs of the RDH and district. The majority of participants felt that the funding of PSOS was feasible because of its financial benefits. General specialties such as general surgery were the most feasible to fund, rather than sub-specialties. It was too costly for an individual sub-district or RDH to employ an array of LPS. A single, PSOS specialist service should rather be utilised by more than one sub-district, and the other sub-districts focus on employing a different LPS tailored to the RDH needs. This, however, required patient transportation to other areas in the district or LPS outreach to other sub-districts from their RDH of employment, resulting in additional costs.

Some participants felt that funding for PSOS should be partly or fully funded by the district or regional hospital because of the alleviated burden on the referral hospital. A small number of participants felt that the district budget for LPS should be adjusted based on the productivity of the PSOS. One participant felt that PSOS needed to be reassessed regularly to ensure that it remained feasible.

Continuing professional development points required time and money for LPS to accumulate in private practice and some private specialists felt that accumulating CPD points from PSOS offered sufficient payment. Several participants felt that with National Health Insurance on the horizon, funding allocations may change and funding PSOS may become a priority.

#### Resource and infrastructure requirements

The majority of participants did not feel that the additional burden of PSOS on RDH resources negatively impacted the feasibility of PSOS. They felt that LPS adapted well to the available resources, were flexible when formulating management plans, and were able to come up with alternative drugs if necessary. Some LPS brought equipment from their private practice or from the regional hospital. Private specialist outreach and support highlighted inadequacies in equipment and drug supplies at the RDH, which allowed for improvements to take place. These improvements had long-term benefits for the RDH and patients.

Resource funding was considered by a few participants to be, in part, the responsibility of the district. This was because of the beneficial effects of PSOS on the district as a whole. Some specialised medications for PSOS potentially hampered feasibility. Most specialised medications were accessible through motivation, but incured additional administrative and financial cost to the pharmacy department. Resource acquisition was most feasible if multiple patients benefitted in long term. Some resource acquisition was achieved by engagement with local welfare organisations, namely ‘Rotary’ and ‘Lions club’.

Consultation rooms and theatre facilities were the only infrastructure requirements for the majority of PSOS.

However, infrastructure requirements depended on the specialty. Some participants felt that an additional theatre for emergencies and doctors competent at performing anaesthetics were also basic requirements. Basic RDH infrastructure should be in place before additional infrastucture specific for PSOS are considered. The regional hospital or district office should partially fund infrastructure improvements for PSOS, if needed:

‘I think it depends on the specialist and what your expectations are of them. I mean if you’re going to send a dermatologist to a clinic, they need much less equipment. If you’re going to send a surgeon to somewhere they might need more equipment, but I think the basics should be in place.’ (Medical officer 3)

#### Implementation of private specialist outreach and support in other rural district hospitals

Private specialist outreach and support was considered to be feasible and practical for implementation in RDHs where PSOS did not exist. However, several important factors needed consideration prior to PSOS implementation. These included assessment of the needs of the RDH, referral hospital and district, assessment of the available LPS in the district, ensuring PSOS does not significantly detract from the primary functions of the RDH, adequate RDH infrastructure, and ensuring that patient safety is maintained. Patient safety was mentioned with reference to appropriate peri-operative care:

‘Taking into consideration time, money, staff availability, transport issues and general good relationships and job satisfaction, my overall feeling is that it is practical and it is feasible.’ (Medical officer 1)

## Discussion

The key findings of this research were grouped into four major themes, namely the roles, effects, sustainability and feasibility of PSOS.

### Roles of local, private specialists

Outpatient care, ward rounds, theatre work, consultation services and training were clinical services provided by LPS. Outpatient, theatre and telephonic consultation services were the most important LPS clinical consultant roles. The clinical services offered by the LPS varied and were dependent on the LPS and the needs of the RDH. Communication with LPS was easier than with public counterparts.^2,9^ As found elsewhere, improved communication with LPS resulted in improved patient management and contributed to the value of PSOS.^2,9^

Multifaceted specialist outreach focuses on capacity building and improving service delivery.^7^ Capacity building was observed, however, to be lacking in public specialist outreach. Local, private specialists performing PSOS were found to have a greater desire to teach, have more enthusiasm for and a greater sense of ownership of rural outreach services compared with their public counterparts.^5^ Skills transfer and capacity building contributed to cultivating sustainable outreach service delivery.^5^

Competency of RDH medical practitioners was improved unanimously, through skills transfer and capacity building.^2,9^ In addition, the positive effects of PSOS skills transfer were felt beyond the sub-district of PSOS. The RDH PSOS ‘champions’ or liaisons were the main beneficiaries of skills transfer and it enhanced the efficiency of the PSOS clinical service programmes, as has also been described before.^9^ An improved competency of RDH medical practitioners, and its widely felt effects greatly contributed to the value of PSOS.

Local, private specialists were not involved in clinical governance at the RDH, which is a role that needs further research, particularly as this is one of the key components of district health districts.

### Effects of private specialist outreach and support

Improved access to services contributed greatly to the value of PSOS. Previous research has shown that PSOS assisted with improved geographic access to specialist care and more timely services.^4,5,7,8,9,10^ This added value to the patients in the local community, with less financial losses, improved quality of life, reduction in loss to follow up, and reduced morbidity and mortality.^4,5,7,8,9,10^ In addition, improved access to care resulted in a reduced need for patient transfer and a positive effect outside the sub-district. Private specialist outreach and support allowed decentralisation of specialist care from larger referral centres. In line with previous work, this research found an alleviated burden on referral centres.^4,5,8^ This included reduced theatre time, bed and financial pressures.^4,5,8^ The availability of RDH healthcare providers was not considered to be negatively affected by PSOS, but PSOS did place an additional burden on the RDH because of the additional services provided. The value of PSOS to the local community and to the district was supported by improved access and timeliness of care, improved competency of RDH medical practitioners, positive effects outside the sub-district, improved coordination, comprehensiveness and continuity of care, and an improved attitude of RDH staff.

### Sustainability of private specialist outreach and support

Multiple benefits existed for LPS involved in PSOS, which contributed to a sustainable public–private relationship. In keeping with findings in the literature, PSOS enabled specialists to widen their scope of practice by exposure to stimulating pathology.^3,5^ In addition, PSOS created a space where LPS could respond to community health needs and provide multifaceted healthcare as part of the RDH team.^3,5^ Remunerated LPS provided a more sustainable service than their pro bono counterparts. However, the sustainability of a PSOS service, for both employed and pro bono LPS, depended mostly on their reasons for involvement in PSOS.

The engagement of the RDH managers with the PSOS strengthened the public–private partnerships, which improved sustainability. Public specialist outreach was more sustainable than PSOS, with the exception being during the COVID-19 pandemic, where public specialist outreach was halted.

### Feasibility of private specialist outreach and support

Many financial benefits of PSOS to the RDH, referral hospital, district and province were identified. It was felt that the funding of PSOS was feasible because of these financial benefits and should ideally be the responsibility of the district. The value of PSOS outweighed the additional burden of PSOS on RDH resources and infrastructure. Private specialist outreach and support was considered to be feasible and practical for implementation in RDHs where PSOS did not exist. However, several important factors needed consideration prior to PSOS implementation. These included assessment of the needs of the RDH, referral hospital and district; assessment of the available LPS in the district; ensuring PSOS does not significantly detract from the primary functions of the RDH; adequate RDH infrastructure; and ensuring that patient safety is maintained.

### Limitations

This was a qualitative study based on the perceived experiences of purposively selected individuals in a particular context and the results may not be generalisable. Perhaps the fact that the study setting was a coastal town regarded as an excellent living environment influenced private specialists to locate their practices there. However, some of the themes that emerged could be applicable to similar contexts elsewhere. The principal researcher worked within the study setting, which may have influenced the results. Potential bias was minimised through ensuring trustworthiness of the data and reflexive practice of the researcher.

### Reflexive statement

The principal researcher is a medical doctor who works within the context of the study setting. He is familiar with the whole healthcare team and lives in the community that is served by the hospital. While the researcher recognised the risk of bias, it was also accepted as an advantage to have in-depth understanding of emerging themes. Potential biases were examined following a mock interview of the principal researcher by one of the research supervisors, using the interview guide. Through regular discussions with the research supervisors, the researcher remained aware of and addressed potential biases.

## Conclusion

The aim of this research was to explore the perceived contribution of PSOS at a RDH in South Africa. Private specialist outreach and support services were sustainable, feasible and had positive effects in and beyond the sub-district. The value of PSOS was supported by improved access to specialist services, improved competency of RDH medical practitioners, positive effects outside the sub-district and enhanced patient care. Private specialist outreach and support was, however, associated with an increased burden on local resources and required basic infrastructure to function effectively. Implementation of PSOS in similar contexts where no PSOS exists is feasible, but requires consideration of several factors.

## Figures and Tables

**FIGURE 1-A1:**
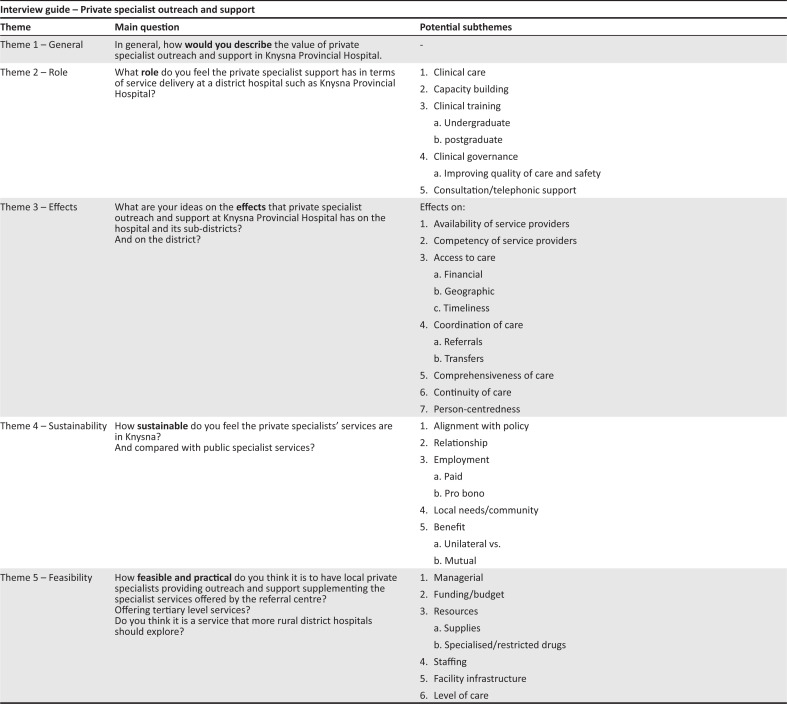
Interview questionnaire.
